# GCPII Inhibition Promotes Remyelination after Peripheral Nerve Injury in Aged Mice

**DOI:** 10.3390/ijms25136893

**Published:** 2024-06-23

**Authors:** Yu Su, Meixiang Huang, Ajit G. Thomas, John Maragakis, Kaitlyn D. J. Huizar, Yuxin Zheng, Ying Wu, Mohamed H. Farah, Barbara S. Slusher

**Affiliations:** 1 Johns Hopkins Drug Discovery, Johns Hopkins University School of Medicine, Baltimore, MD 21205, USA; ysu46@jh.edu (Y.S.); mhuang52@jh.edu (M.H.); ajit.thomas@jhmi.edu (A.G.T.); jmaraga1@jhu.edu (J.M.); khuizar1@jhmi.edu (K.D.J.H.); yzhen124@jh.edu (Y.Z.); ywu58@jhmi.edu (Y.W.); 2 Department of Neurology, Johns Hopkins University School of Medicine, Baltimore, MD 21205, USA; 3 Department of Pharmacology and Molecular Sciences, Johns Hopkins University School of Medicine, Baltimore, MD 21205, USA; 4 Department of Medicine, Johns Hopkins University School of Medicine, Baltimore, MD 21205, USA; 5 Department of Oncology, Johns Hopkins University School of Medicine, Baltimore, MD 21205, USA; 6 Department of Psychiatry and Behavioral Science, Johns Hopkins University School of Medicine, Baltimore, MD 21205, USA; 7 Department of Neuroscience, Johns Hopkins University School of Medicine, Baltimore, MD 21205, USA

**Keywords:** aging, PNI, GCPII, remyelination, Schwann cells, macrophages

## Abstract

Peripheral nerve injuries (PNIs) represent a significant clinical challenge, particularly in elderly populations where axonal remyelination and regeneration are impaired. Developing therapies to enhance these processes is crucial for improving PNI repair outcomes. Glutamate carboxypeptidase II (GCPII) is a neuropeptidase that plays a pivotal role in modulating glutamate signaling through its enzymatic cleavage of the abundant neuropeptide N-acetyl aspartyl glutamate (NAAG) to liberate glutamate. Within the PNS, GCPII is expressed in Schwann cells and activated macrophages, and its expression is amplified with aging. In this study, we explored the therapeutic potential of inhibiting GCPII activity following PNI. We report significant GCPII protein and activity upregulation following PNI, which was normalized by the potent and selective GCPII inhibitor 2-(phosphonomethyl)-pentanedioic acid (2-PMPA). In vitro, 2-PMPA robustly enhanced myelination in dorsal root ganglion (DRG) explants. In vivo, using a sciatic nerve crush injury model in aged mice, 2-PMPA accelerated remyelination, as evidenced by increased myelin sheath thickness and higher numbers of remyelinated axons. These findings suggest that GCPII inhibition may be a promising therapeutic strategy to enhance remyelination and potentially improve functional recovery after PNI, which is especially relevant in elderly PNI patients where this process is compromised.

## 1. Introduction

PNIs affect approximately 3% of trauma patients resulting in symptoms ranging from muscle denervation and locomotor dysfunction to pain and sensory loss [1,2]. Despite the inherent regenerative capacity of peripheral nerves, this ability declines significantly with age [3,4,5,6]. Although enhancing myelination and nerve regeneration after PNI is an active area of research [7,8,9], there has been a lack of effective therapies developed to date. Research indicates that the diminished regenerative potential observed during aging is not intrinsic to neurons but rather stems from compromised repair mechanisms involving Schwann cells [6,10,11] and macrophage infiltration within the distal segment [12,13,14,15].

Schwann cells are central to nerve regeneration, promoting axonal regrowth and remyelination after injury [16,17]. These glial cells coordinate this process through a comprehensive response involving de-differentiation, proliferation, reformation of myelin sheaths, and secretion of neurotrophic factors [18]. After injury, Schwann cells undergo a transformation from a myelin-forming state to a repair phenotype [18,19]. Simultaneously, there’s a downregulation of myelin markers like P0 and S100 and an upregulation of repair-related markers like GFAP and others that support axonal regeneration and myelin breakdown [18,20]. However, aging significantly impacts this Schwann cell response, leading to decreased de-differentiation potential, impaired remyelination gene programs, and reduced trophic support [11,21]. In addition to Schwann cells, macrophages also play an essential role in PNI [12,22]. Following nerve injury, macrophages are rapidly activated and recruited to the site of damage, performing crucial functions through phagocytosis of myelin debris and secretion of cytokines, chemokines, and growth factors that support Schwann cell de-differentiation, proliferation, and remyelination [18,23,24]. Recent studies have shown that the recruitment and activation of macrophages are tightly regulated by signaling molecules released from injured neurons and Schwann cells [25,26]. However, the efficiency of these processes declines with age, leading to impaired clearance of myelin debris and a prolonged inflammatory response, which can hinder remyelination. As a result, regenerated axons in aged nerves frequently remain poorly remyelinated or demyelinated, hindering the re-establishment of saltatory conduction and trophic interactions that are crucial for axon survival and maturation. This incomplete remyelination ultimately leads to persistent functional deficits, including impaired motor coordination and reduced sensory perception.

This age-related decline in the pro-regenerative functions of Schwann cells and activated macrophages represents a major barrier to achieving nerve repair outcomes, particularly in the elderly population. GCPII is a membrane-bound enzyme that cleaves the abundant neuropeptide NAAG into N-acetyl aspartate (NAA) and glutamate [27,28,29]. Recently GCPII was found to be upregulated in Schwann cells and activated macrophages after nerve injury [30,31,32,33]. Previous studies have also demonstrated that GCPII expression and activity increase following brain injury, and its inhibition improves cognitive impairment [34,35,36]. In the peripheral nervous system (PNS), pharmacological inhibition of GCPII has demonstrated the preservation of nerve structure and function in models of chemotherapy-induced neurotoxicity and diabetic neuropathy [37,38]. However, the role of GCPII in regulating remyelination, particularly in the context of aging, remains largely unexplored.

Given the upregulation of GCPII with aging and inflammation, and the promising effects of GCPII inhibition in neurological and inflammatory conditions, this study investigated the therapeutic potential of inhibiting GCPII after PNI, with a specific focus on addressing the age-related remyelination deficit. Utilizing the potent and selective GCPII inhibitor 2-PMPA, we evaluated its effects in vitro on Schwann cell myelination in DRG explants and in vivo, using a sciatic nerve crush model in aged mice. Our findings elucidate a novel therapeutic utility for GCPII inhibition in treating PNIs, especially for elderly populations exhibiting impaired remyelination capacity.

## 2. Results

### 2.1. GCPII Activity and Expression Are Elevated in Peripheral Nerves after Injury

**Figure 1:**
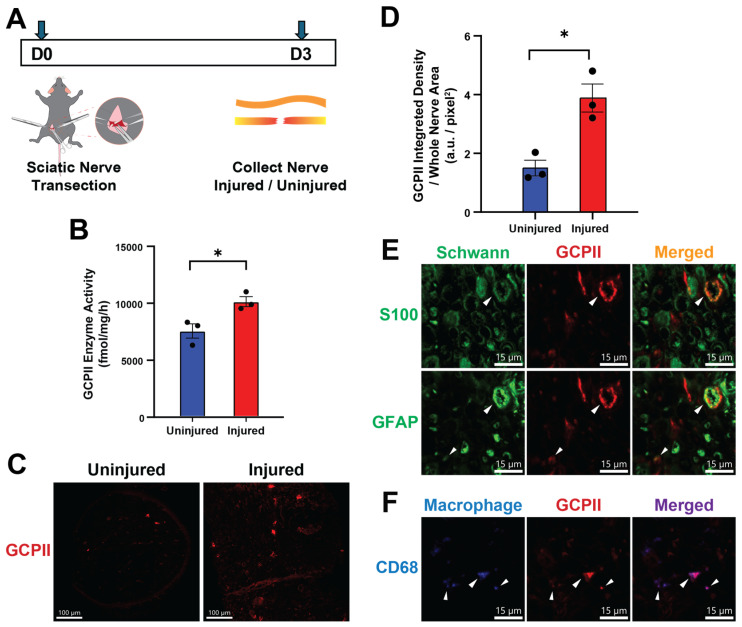
GCPII enzymatic activity and expression increased following peripheral nerve injury. (**A**) Experimental design schematic: 3-month-old mice underwent left sciatic nerve transection, and 3 days later, both the transected nerve and the contralateral uninjured nerve were collected for enzymatic activity and expression analysis. (**B**) GCPII enzymatic activity was significantly higher in the injured nerve compared to the uninjured contralateral nerve (10,047 ± 440 vs. 7464 ± 626 fmol/mg/h; *p* < 0.05, *n* = 3). (**C**) GCPII immunofluorescent staining in the injured nerve was more abundant than in the uninjured nerve. Scale bar in images indicates 100 μm (**D**) GCPII expression was quantified by calculating the integrated immunofluorescent density divided by the nerve area, analyzed using Image J 2.14.0 (1.50 ± 0.27 vs. 3.88 ± 0.48 arbitrary units/pixel2; *p* < 0.05, *n* = 3). (**E**) GCPII (red) was co-localized with Schwann cell markers S100 and GFAP (green) in the injured nerve (arrows indicating colocalization). Scale bar in images indicates 15 μm. (**F**) GCPII (red) was colocalized with the macrophage marker CD68 (blue) in the injured nerve (arrows indicating colocalization). Scale bar in images indicates 15 μm. Statistics were performed using unpaired Student’s *t*-tests. Bars represent mean ± SEM. * *p* < 0.05, *n* = 3.

We first evaluated GCPII activity and expression in a mouse peripheral nerve crush model by both enzymatic assay and immunostaining (Figure 1A). Three days post-transection in the sciatic nerve, GCPII activity significantly increased in the distal segment of the injured nerve compared to the uninjured contralateral side (10,047 ± 440 vs. 7464 ± 626 fmol/mg/h; *p* < 0.05) (Figure 1B). Elevated GCPII expression in the injured nerve was also confirmed by immunofluorescent staining. Cross-sections of the injured nerve showed a higher GCPII staining signal than the uninjured side (Figure 1C), with quantification revealing a significant increase in integrated immunofluorescent density (1.50 ± 0.15 vs. 3.88 ± 0.28 a.u./pixel^2^; *p* < 0.05) (Figure 1D).

To identify the cellular sources of elevated GCPII expression in the nerve after injury, we stained the samples using Schwann cell (S100, GFAP) and macrophage (CD68) markers. At 3 days post-denervation, the distal nerve segments exhibited axonal degeneration and infiltration of macrophages. GCPII immunoreactivity was found to co-localize with markers of Schwann cells, especially GFAP, which is a marker of repair Schwann cells. Furthermore, many cells expressing GCPII were found to co-localize with macrophage markers, indicating that both Schwann cells and infiltrating macrophages contribute to the increased levels of GCPII.

### 2.2. GCPII Inhibition with 2-PMPA Promotes Myelination In Vitro

**Figure 2:**
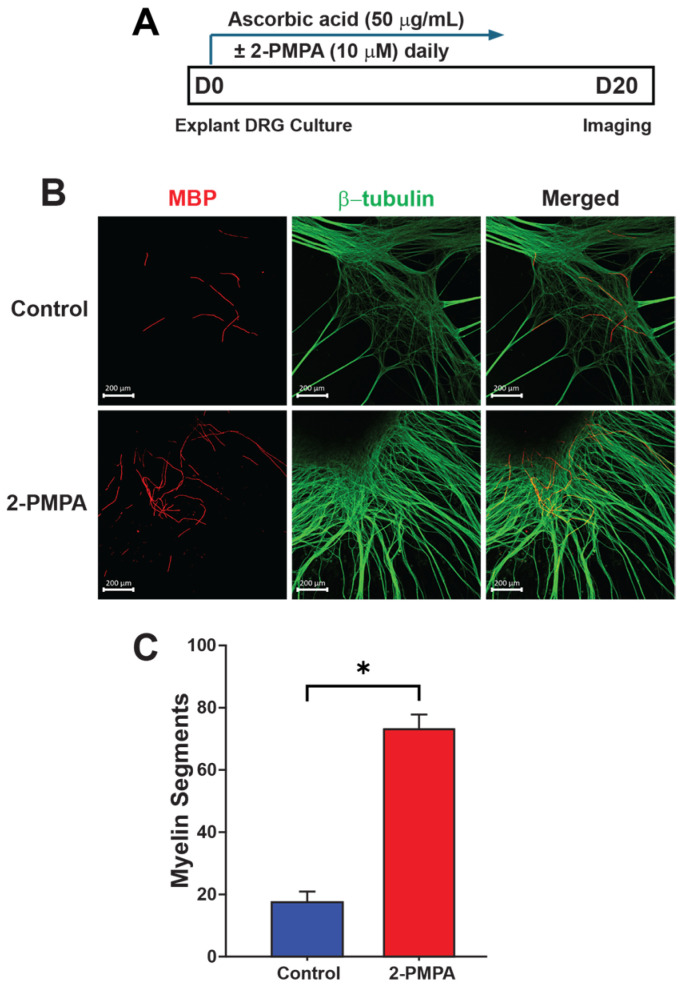
GCPII inhibition enhances myelination in vitro in DRG explant cultures. (**A**) Experimental design schematic. DRGs were extracted from postnatal mice on days 4–5 and cultured in dishes with ascorbic acid to stimulate myelination. GCPII inhibitor 2-PMPA (10 μM) or vehicle was added to the culture daily. (**B**) Representative images of DRG explants with immunofluorescent staining for MBP (red) and β-tubulin (green). Scale bar in images indicates 200 μm. (**C**) Quantitative analysis of myelin segments showed significantly higher myelin in 2-PMPA-treated compared to the vehicle-treated cultures (17.67 ± 3.48 vs. 72.67 ± 4.37; *p* < 0.05, *n* = 3). Statistics were performed using unpaired Student’s *t*-tests. Bars represent mean ± SEM. * *p* < 0.05, *n* = 3.

Given the previously reported effects of GCPII inhibition in preserving nerve structure and function in models of chemotherapy-induced neurotoxicity and diabetic neuropathy [37,38] and the significant increase in GCPII expression in Schwann cells following nerve injury, we next aimed to investigate whether inhibiting GCPII would benefit nerve regeneration, specifically focusing on myelination. To assess the potential for GCPII inhibition to promote myelination in vitro, we used a DRG explant containing DRG neurons and Schwann cells. DRG explants from postnatal mice were cultured for up to 10 days in the presence of either the GCPII inhibitor 2-PMPA (10 μM) or vehicle. Ascorbic acid was supplemented to stimulate Schwann cell myelination (Figure 2A). Immunofluorescence staining for myelin basic protein (MBP) and β-tubulin, followed by confocal microscopy analysis, revealed a significantly higher number of myelin segments in DRG explants treated with 2-PMPA compared to the vehicle control (Figure 2B). This was evidenced by increased MBP and myelin segment formation along axons in 2-PMPA-treated cultures (17.67 ± 3.48 vs. 72.80 ± 4.58; *p* < 0.05) (Figure 2C). These findings suggest that pharmacological inhibition of GCPII activity with 2-PMPA promotes myelin formation.

### 2.3. GCPII Inhibition Accelerates Remyelination after PNI in 3-Month-Old Mice

**Figure 3:**
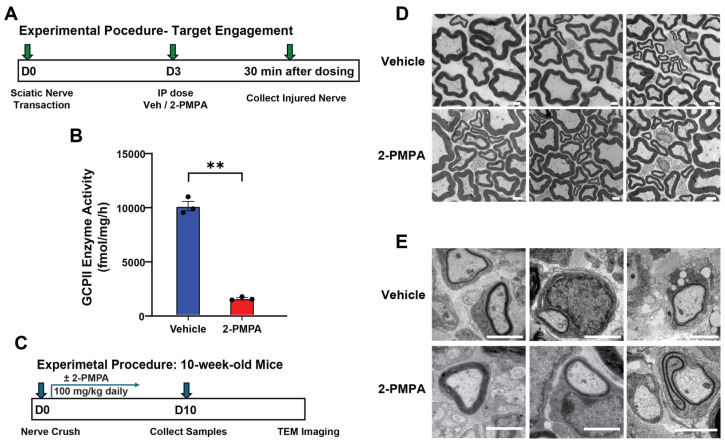
GCPII inhibition enhances remyelination in mice following PNI. (**A**) Experimental design schematic: 3-month-old mice underwent left-side sciatic nerve transection. Three days post-transection, mice were dosed IP with 2-PMPA or vehicle and 30 min later nerves from both groups were collected for GCPII activity quantification. (**B**) The 2-PMPA treatment decreased GCPII activity in the injured nerves by 85% (10,047 ± 440 vs. 1504 ± 89 fmol/mg/h; *p* < 0.01, *n* = 3), demonstrating strong target engagement. (**C**) Experimental design schematic: 3-month-old mice underwent sciatic nerve crush injury followed by daily 2-PMPA (100 mg/kg, IP) or vehicle for 10 days. Nerves were then collected for TEM imaging. (**D**) Representative TEM images of uninjured axon structures from mice treated with vehicle or 2-PMPA showing that 2-PMPA did not alter axon or myelination morphology in healthy nerves. Scale bar in images indicates 1 μm. (**E**) Representative TEM images of crushed axon structures showing that 2-PMPA treatment enhanced remyelination after crush injury. Scale bar in images indicates 1 μm. Statistics were performed using unpaired Student’s *t*-tests. Bars represent mean ± SEM. ** *p* < 0.01, *n* = 3.

Given these promising in vitro data, we proceeded to in vivo studies to further investigate the effects of GCPII inhibition on remyelination. We chose to initially test 2-PMPA at a daily intraperitoneal (IP) dose of 100 mg/kg based on previous pharmacokinetics studies [39]. To confirm target engagement using this dose in our PNI model, we measured GCPII activity in the peripheral nerve 30 min following a 100 mg/kg IP dose (Figure 3A,B). The 2-PMPA significantly attenuated the injury-induced elevation of GCPII activity (10,047 ± 440 vs. 1504 ± 89 fmol/mg/h; *p* < 0.01), demonstrating robust target engagement.

With the confirmed inhibition, we next evaluated the effect of 2-PMPA on remyelination following PNI (Figure 3C). Following nerve crush, mice received either IP 2-PMPA or vehicle for 10 days. At the end of the treatment period, both uninjured nerves and distal segments of the injured nerves were collected for analysis. Nerve morphology and remyelination were examined in healthy nerves and 4 mm distal to the crush site in the injured nerves using transmission electron microscopy (TEM). As shown in Figure 3D, treatment with 2-PMPA did not induce morphological changes in uninjured nerves compared to vehicle-treated controls, indicating no adverse effects on nerve structure under normal physiological conditions. In the injured nerve, however, 2-PMPA treatment had a significant effect on accelerating remyelination compared to vehicle controls, as seen by an increase in myelin sheath area (Figure 3E).

### 2.4. GCPII Inhibition Enhances Remyelination after PNI in 20-Month-Old Mice

**Figure 4:**
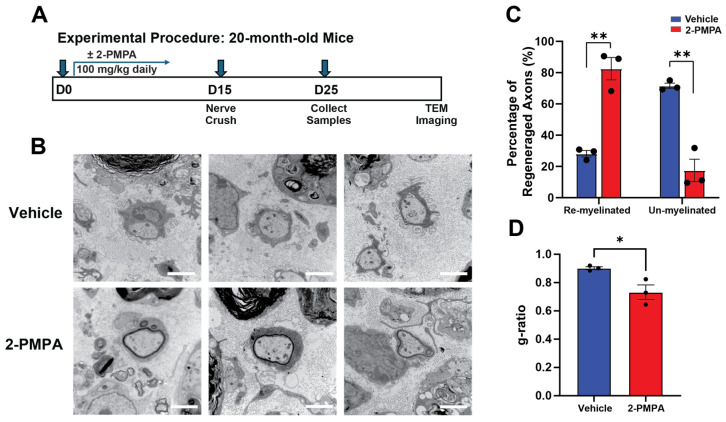
GCPII inhibition enhances remyelination in aged mice following PNI. (**A**) Experimental design schematic: 20-month-old mice were treated with vehicle or 2-PMPA for 15 days, and then underwent sciatic nerve crush injury. Treatment with vehicle or 2-PMPA continued for an additional 10 days before mice were sacrificed and their nerves collected for TEM. (**B**) Representative TEM images of crushed axon structures from mice treated with vehicle or 2-PMPA showing that 2-PMPA treatment enhanced remyelination in aged mice after crush injury. Scale bar in images indicates 1 μm. (**C**) The 2-PMPA-treated group showed 82.5 ± 7.2% remyelination, while the vehicle-treated group showed 28.1 ± 2.1% remyelination (21–27 axons with a diameter above 1 μm were analyzed per nerve; *p* < 0.01, *n* = 3). (**D**) The 2-PMPA-treated group showed a g-ratio of 0.73 ± 0.05, while the vehicle-treated group showed a g-ratio of 0.90 ± 0.01 (*p* < 0.05). Statistics were performed using unpaired Student’s *t*-tests. Bars represent mean ± SEM. * *p* < 0.05, ** *p* < 0.01, *n* = 3.

The improved myelination of DRG explants and remyelination in 3-month-old mice following PNI encouraged us to investigate PNI recovery in aged mice, where impaired recovery is observed. We pre-treated the 20-month-old mice with either 2-PMPA or vehicle for 15 days before sciatic nerve crush surgery. After nerve crush, the aged mice continued to receive either 2-PMPA or vehicle for another 10 days (Figure 4A).

Histological analysis at 10 days post-injury revealed that although the remyelination was delayed in the aged mice, the 2-PMPA-treated group exhibited significantly more remyelination of axons compared to the vehicle-treated controls (Figure 4B,C). Additionally, quantifying g-ratios further demonstrated enhanced myelin sheath thickness relative to axon diameter following 2-PMPA administration (0.73 ± 0.03 vs. 0.90 ± 0.01; *p* < 0.05) (Figure 4D), representing a significant improvement in remyelination compared to the untreated group, despite not being fully recovered to normal levels. Combining the data from Figure 4C,D indicates that GCPII inhibition is more efficient at initiating remyelination rather than increasing remyelination on individual axons within the examined time points. These results demonstrate that pharmacological inhibition of GCPII with 2-PMPA is effective in enhancing repair after nerve injury, even in an aged context where endogenous remyelination capacity is impaired.

## 3. Discussion

In this study, we investigated the therapeutic potential of GCPII inhibition for enhancing remyelination after PNI, with a focus on improving the age-related deficit in remyelination capacity. We first report an elevation of GCPII following PNI in both Schwann cells and macrophages. Using the potent inhibitor 2-PMPA, we showed that GCPII inhibition could robustly enhance myelination in DRG explants. Subsequently, in both young (3-month-old) and aged (20-month-old) mice undergoing sciatic nerve crush, we found that GCPII inhibition could significantly accelerate remyelination. These results highlight GCPII inhibition as a promising therapeutic strategy to enhance nerve remyelination and regeneration after injury, particularly for older individuals with reduced remyelination capacity.

Schwann cells play a pivotal role in nerve regeneration after PNI, with mature Schwann cells being essential for this process [40,41,42]. Following PNI, both myelin and non-myelin (Remak) Schwann cells transform into repair Schwann cells by de-differentiating from the myelin-forming state and activating repair phenotypes. This transition includes upregulating trophic factors and surface proteins to support injured neurons, initiating autophagy for myelin breakdown, and recruiting macrophages for immune response [18,43]. Specifically, this process involves the downregulation of myelin-related genes, such as S100 and MBP, and the upregulation of repair markers, such as GFAP, p75 neurotrophin receptor (p75NTR), glial cell line-derived neurotrophic factor (GDNF), and brain-derived neurotrophic factor (BDNF) [18]. The transcription factor c-Jun is crucial in this reprogramming [6,44]. Repair Schwann cells, assisted by infiltrating activated macrophages, efficiently remove myelin debris through autophagy or phagocytosis and form regeneration tracks known as Büngner bands, to guide axons to their targets [43,45,46]. Our results demonstrated that the injured nerve had elevated Schwann cell GCPII expression that was coincident with the transition from myelinating (S100 expressing) to repair (GFAP expressing) Schwann cells (Figure 1E).

Macrophages also play an indispensable role in Schwann cell-mediated nerve regeneration after PNI [12,22]. Macrophages are actively recruited by repair Schwann cells and infiltrate into the injured nerve, where they are critical for the efficient removal of degenerated myelin debris through phagocytosis, creating a positive environment for axonal regrowth [23,24] (Figure 1F). Furthermore, the recruited macrophages secrete cytokines, chemokines, and growth factors that not only promote myelin clearance by Schwann cells, but also support Schwann cell dedifferentiation, proliferation, and remyelination [18,45]. Notably, we found that GCPII is significantly upregulated in both Schwann cells and macrophages following PNI, suggesting that GCPII may modulate the functions of both cell types to facilitate remyelination and regeneration. Previous studies have implicated GCPII in regulating macrophage-induced inflammatory responses [31,32]. Therefore, the remyelination and regeneration-promoting effects of GCPII inhibition observed in our study may be mediated by its actions on both Schwann cell and macrophage populations, cooperatively driving nerve repair after injury. Macrophage GCPII has recently emerged as a potential modulator of peripheral nerve function, as demonstrated using the SOD murine model of Amyotrophic lateral sclerosis (ALS) [32], and its expression has been shown to be elevated under both aging and neuroinflammatory conditions [34,47]. Our data demonstrating that pharmacological inhibition of GCPII with 2-PMPA enhances remyelination following PNI suggests a novel therapeutic strategy to promote regeneration. The most exciting data was the observation that GCPII inhibition could overcome age-related deficits in remyelination following PNI, given that aging is known to significantly impair the endogenous regenerative program of Schwann cells [11,48]. In aged mice, Schwann cells show diminished plasticity and are slower to activate transcriptional repair programs, which results in impaired de-differentiation and myelin clearance [49]. The delay in the recruitment of macrophages into the injury site further slows the regeneration process. Furthermore, impaired activation of c-Jun and other growth factors with aging also diminishes axonal regeneration [11]. Our results indicate GCPII inhibition as a promising therapeutic target to rejuvenate remyelination following PNI, even with aging.

The mechanisms by which GCPII inhibition promotes remyelination and regeneration remain to be fully elucidated. However, based on our data and other studies on glutamate’s inhibitory role in myelination [50,51], we hypothesize that GCPII inhibition could benefit remyelination after PNI through two primary mechanisms. First, the elevated GCPII levels in Schwann cells post-injury indicate that its inhibition enhances their reprogramming to the repair phenotype, promoting myelin debris removal. Second, GCPII inhibition has been shown to reduce macrophage-related inflammation in other conditions, potentially downregulating pro-inflammatory pathways and shifting macrophage activation toward a pro-regenerative state. This environment supports Schwann cell proliferation and differentiation, resulting in improved remyelination.

## 4. Materials and Methods

### 4.1. Animals

Animal procedures performed in this study conformed to the guidelines of, and were approved by, the Johns Hopkins University Animal Care and Use Committee. All animals were housed in the Miller Research Building animal facility at Johns Hopkins. For peripheral nerve injury and GCPII activity experiments, 3-month-old C57BL/6J male mice were used, obtained from Jackson Laboratory. For peripheral nerve injury and nerve morphology analysis experiments, 20-month-old male and female C57BL/6J mice were acquired from Charles River (Rockville, MD, USA). The mice were placed into different experimental conditions by random assignment. For the drug dosing regimen, the mice were injected with 2-PMPA at 100 mg/kg or 1× HEPES buffer (7365-45-9, San Diego, CA, USA) as vehicle intraperitoneally daily.

### 4.2. Sciatic Nerve Injury

The mouse sciatic nerve injury model was described previously [44,52]. Briefly, to induce peripheral nerve injury, mice were completely anesthetized with 1–2% isoflurane and prepped for surgery underneath a dissection stereo microscope. The left hindquarter was shaved and cleaned with 70% ethanol. An incision was made to expose the sciatic nerve, and forceps were used to gently separate the intact sciatic nerve from the surrounding musculature. A 10-0 non-absorbable black suture was passed underneath the nerve. Using the tips of the forceps, the same nerve segment was crushed 3 times for 20 s each, rendering the crushed portion translucent. The suture was then loosely tied around the nerve, serving as a marker of the injury site rather than for constriction. The nerve was then carefully replaced, and the skin incision was closed with a staple (F.S.T, 12032-09, Foster City, CA, USA). 1 mg/kg of Buprenorphine ER Lab (Wedgewood Pharmacy, Swedesboro, NJ, USA) was injected subdermally as an analgesic.

In sciatic nerve transection procedures, after analgesia was administered in the same way as detailed above, the nerve was completely severed at the same mid-thigh location. The resulting cut ends were separated and turned away from one another to prevent the axonal reconnecting of the two stumps. Following this, the incision was closed. In transection experiments, the severed nerve, as well as the intact contralateral side, were collected 3 days after transection for enzymatic analysis.

### 4.3. GCPII Enzyme Activity Assay

GCPII activity measurements were carried out based on modifications of previously published protocols [39,53]. To perform the GCPII enzyme activity assay in mouse nerve tissue, 3-month-old C57BL/6J mice were subjected to sciatic nerve transection. Three days post-transection, the animals were euthanized, and both the injured and uninjured sciatic nerves were dissected, snap-frozen, and stored at −80 °C until GCPII enzyme activity measurements were performed. To increase sample volume, sciatic nerves from 3 mice were pooled together to form one sample, resulting in a total of 9 mice per group to achieve *n* = 3. Briefly, nerves were homogenized in ice-cold Tris buffer (40 mM, pH 7.5 at 7.5 mg/mL) containing protease inhibitors using Omni’s Bead Ruptor Elite (2 mL reinforced polypropylene tubes filled with six 2.8 mm ceramic beads, at 6 m/s for 20 s, 3 cycles, 30 s between cycles, and a temperature of −1 to 0 °C) and further sonicated using Kontes’ Micro Ultrasonic Cell Disrupter (three pulses of 30 s duration, 30 s between pulses, and on ice). The resulting homogenates were spun down (16,000× *g* for 2 min at 4 °C), and the supernatants were collected for both GCPII activity and total protein analysis. The GCPII reaction was initiated upon the addition of pre-warmed (37 °C) cobalt chloride (1 mM) and ^3^H-NAAG (0.04 µM, 49.79 mCi/µmol). The reactions were carried out in 50 µL reaction volumes in 96-well microplates for 180 min at 37 °C. Reactions were terminated with ice-cold sodium phosphate buffer (100 mM, pH 7) with 1 mM EDTA. A total of 96 well spin columns packed with anion exchange resin were used to separate the substrate and the reaction product. The reaction product, [^3^H]-glutamate, was eluted with 1 M formic acid, and analyzed for radioactivity. Finally, the total protein was quantified per the manufacturer’s instructions using the Bio-Rad DC Protein Assay kit (5000111, Hercules, CA, USA), and GCPII activity data were calculated as fmoles of NAAG hydrolysis per mg protein per hour (fmol/mg/h).

### 4.4. Immunofluorescent Staining

The nerve samples for immunohistochemistry were prepared and processed as previously described, with minor modifications [54,55,56]. Briefly, the nerves were dissected out and immersed in 4% PFA overnight, then washed with 1× PBS and dehydrated in 30% sucrose in 1× PBS before being embedded in Tissue-Tek^®^ O.C.T. compounds (Sakura Finetek, #4583, Torrance, CA, USA). Samples were cryo-sectioned at 15 μm. To decrease non-specific binding, the samples were first blocked with 5% normal goat serum in 1× PBS for 1 h at room temperature after being permeabilized with 0.2% TrixonX-100 for 10 min, then blocked with M.O.M Mouse IgG Blocking Reagent (Vector Laboratories, MKB-2213-1, Newark, CA, USA) for 1 h at room temperature. Depending on the target proteins of the experiment, a primary antibody was chosen (Table 1), and the samples were incubated with it overnight at 4 °C. Following this, samples were washed 3 times (5 min each) in 1× PBS and incubated with appropriate secondary antibodies (Table 1) for 1 h at room temperature. After washing 3 times (5 min each) in 1× PBS again, the slides were mounted (ProLong™ Gold Antifade Mountant, Invitrogen, P36930), and coverslipped for imaging under Andor BC43 confocal microscopy (Oxford Instruments, Belfast, UK), and analyzed with ImageJ 2.14.0.

### 4.5. Dorsal Root Ganglia Explants

DRG explant culture was performed with modifications from previously published methods [57,58,59]. C57BL/6 male mice were obtained from Jackson Laboratory. DRGs were dissected on postnatal days 4–5 (P4–P5) [54], then cultured in a Neurobasal medium supplemented with 2% B27. Ascorbic acid at a concentration of 50 μg/mL was added daily to stimulate myelination by enhancing the synthesis of extracellular matrix and basal lamina assembly in Schwann cells. In the experimental conditions, vehicle or 10 μM 2-PMPA was added to the media daily. Each condition contained eight or more DRG explants. The DRG explants were examined at 5 and 10 days under fluorescent microscopy to visualize axonal outgrowth and remyelination. Samples were stained with β-tubulin and MBP to highlight neurofilament and MBP, respectively. The myelination of axons was quantified by counting individual myelin segments along axons, defined by their MBP. The total number of newly myelinated segments for each explant was recorded. All DRG culture imaging was performed with a Zeiss confocal microscopy (LSM800, White Plains, NY, USA) using Zen software 3.10.

### 4.6. Morphological Analyses of Nerves

Sciatic nerves were assessed at the EM level for alteration in axonal integrity, as previously described [52,60]. Briefly, sciatic nerve samples were dissected and immediately fixed overnight at 4 °C in 2% glutaraldehyde/2% PFA in 1× PBS, buffered to pH 7.4. After being fixed, the samples were washed in 1× PBS, postfixed in 1% OsO_4_, and embedded in Epon. Samples were prepared for ultrastructural analysis imaging using TEM. Samples for TEM were sectioned to 70 nm thickness using an ultracut ultramicrotome and stained with citrate/uranyl acetate. These sections were examined for remyelination using a Hitachi 7600 transmission electron microscope (Santa Clara, CA, USA). A total of 20–30 axons were analyzed from each animal. The g-ratio of the aged mice was measured and calculated using ImageJ 2.14.0.

### 4.7. Statistical Analysis

Statistical analysis was performed using an unpaired Student’s *t*-test through GraphPad Prism 10. Data presented in the figures are displayed as the mean ± SEM. The significance levels were indicated as ** *p* < 0.01 and * *p* < 0.05.

## 5. Conclusions

In conclusion, for the first time, this study establishes GCPII as a novel regulator of Schwann cell and macrophage function following PNI and highlights its inhibition as a potential therapeutic strategy to enhance repair, particularly in an aging context where endogenous remyelination capacity is compromised. Further investigations, including electrophysiology and gait analysis, are necessary to confirm the functional improvements in the mouse model and validate the role of GCPII inhibition in nerve regeneration.

## Figures and Tables

**Table 1:** List of antibodies.

Target	Primary Antibody	Secondary Antibody
GCPII	GCP-04 (Goat anti-Mouse, 1:250) (Novus, NBP1-45057, Centennial, CO, USA)	647 nm, anti-mouse IgG1 (1:1000) (Invitrogen, A66784, Waltham, MA, USA)
Repair Schwann cells	GFAP (Goat anti-Chicken, 1:250) (Abcam, Ab4674, Waltham, MA, USA)	488 nm, anti-chicken IgG (H + L) (1:1000) (Invitrogen, A11039, Waltham, MA, USA)
Myelin Schwann cells	S100 (Goat anti-Rabbit, 1:25) (DAKO, GA50461-2, Santa Clara, CA, USA)	546 nm, anti-rabbit IgG (H + L) (1:1000) (Invitrogen, A11035, Waltham, MA, USA)
Macrophages	CD68 (Goat anti-Rat, 1:250) (Invitrogen, PA5-109344, Waltham, MA, USA)	405 nm, anti-rat IgG (H + L) (1:1000) (Invitrogen, A48261, Waltham, MA, USA)

## Data Availability

Data are contained within the article.
